# Supplementary figures and images for: The mitotic spindle protein CKAP2 potently increases formation and stability of microtubules
Source: eLife. 2022 Jan 14;11:e72202. doi: 10.7554/eLife.72202 (PMC8798059; doi:10.7554/eLife.72202)

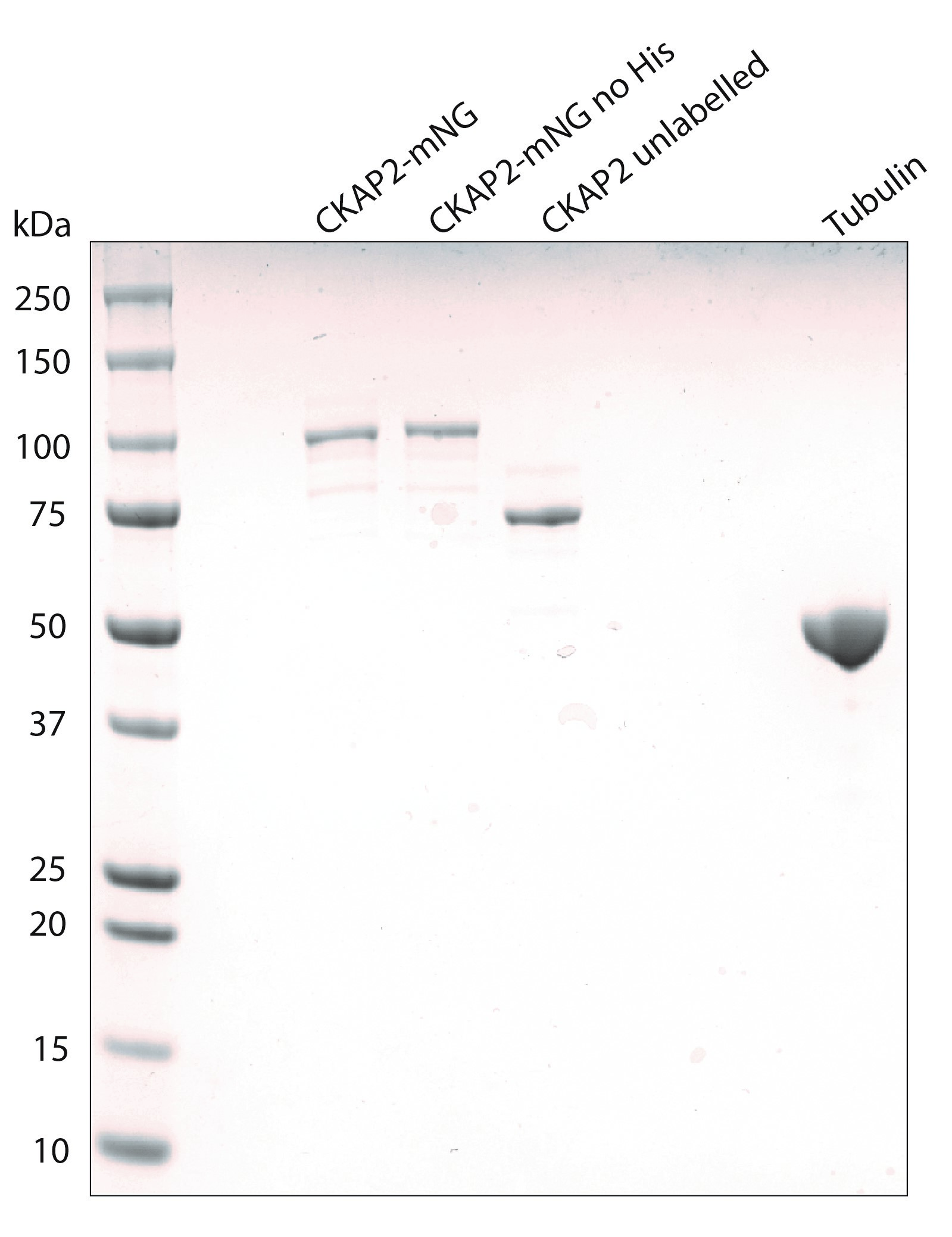

Supplement: Figure 1—source data 1. [file elife-72202-fig1-data1.zip › CKAP2 gel labelled.jpg]

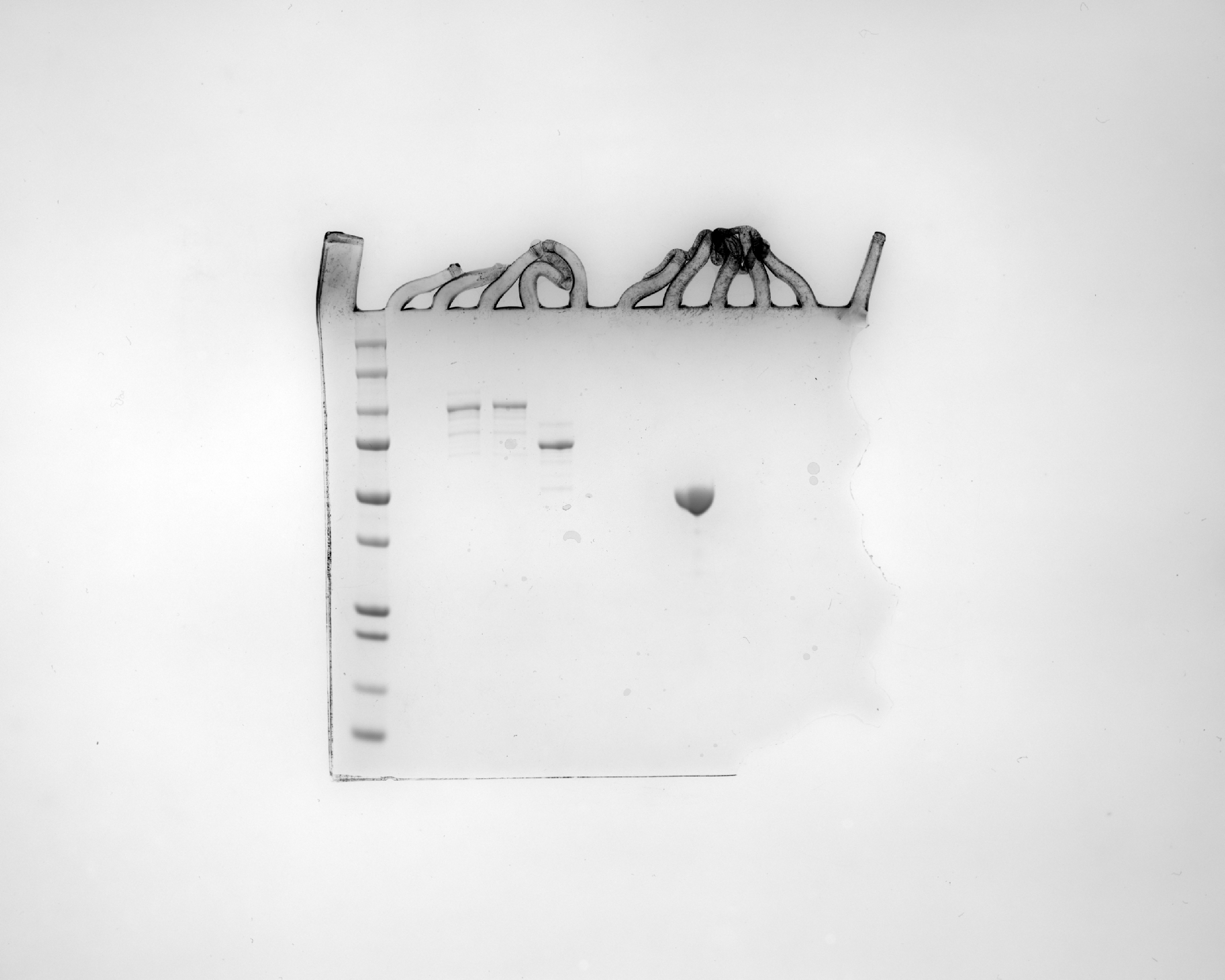

Supplement: Figure 1—source data 1. [file elife-72202-fig1-data1.zip › CKAP2 gel original.jpg]
